# Bacterial Milk Quality along the Value Chain in Smallholder Dairy Production

**DOI:** 10.1155/2022/7967569

**Published:** 2022-09-21

**Authors:** S. Washaya, C. Jakata, M. Tagwira, T. Mupofu

**Affiliations:** ^1^Africa University, College of Health Agriculture and Natural Sciences, Department of Agriculture, P Bag 1320, Mutare, Zimbabwe; ^2^Great Zimbabwe University, Faculty Agriculture and Natural Sciences, Department of Livestock, Wildlife and Fisheries, P. Bag 1235, Masvingo, Zimbabwe

## Abstract

This study aimed to evaluate the microbial quality of raw milk along the milk value chain at Africa University (AU). Eighteen Holstein-Friesian cows were used in this experiment. A total of 270 milk samples were collected for laboratory analysis at three different stages, during milking (DM), from the bulk tank (BT), and at the dining hall (DH), to determine the total bacterial count (TBC), *Escherichia coli*, and *Salmonella enterica*. Samples were cultured in Petri dishes using an appropriate medium for 48 hours. The plate count method was used to determine the quantity of bacteria. Data were analyzed using GLM SPSS. The results indicated that TBC increased (*P* < 0.05) from one site to the next along the value chain, yet it undulates when measured over time. *Escherichia*. *coli and S. enterica* counts increased (*P* < 0.05) at the last site of collection and the highest counts were recorded in week two. In conclusion, the current study indicates the hygiene of the dairy parlor with very low TBC, *E*. *coli,* and *S. enterica* counts during milking and bulk tank storage and that the relationship between TBC and *E. coli* is nonlinear with respect to time.

## 1. Introduction

Milk is an essential source of nutrients to both humans and animals [1]. On average, 87% of milk is on average composed of 87% water and 13% total solids (carbohydrates, fat, proteins, and minerals) contained in a balanced form. These elements are generally digestible for both animals and humans and are essential for body building and maintenance [1]. In context, milk has a complex biochemical composition [1], high water activity, and nutritional value; these serve as an excellent medium for microbial growth and multiplication [2]. These conditions precede the spoilage of milk, whether raw or processed, leading to infection/intoxication in consumers. When milk is synthesized within the mammary gland, it is virtually sterile up until the alveoli of the udder [3]. Beyond this stage, bacterial contamination can occur from three main sources; within or outside the udder, and/or from the surfaces of numerous equipment used for milk handling and storage [5]. In addition, Torkar and Teger [4] reported that animal health, the prevailing environment, milking procedures, and equipment sanitation have a significant influence on the level of microbial contamination. To this end, milk for either human or animal consumption must be free from pathogenic organisms [5]. Unfortunately, a number of potentially pathological microorganisms have been isolated from raw milk and milk products, some of which include *E. coli, Staphylococcus aureus, Salmonella* spp.*, Listeria moncytogens*, and *Brucella abortus* [6]. The major pathogenic bacteria commonly found in milk include *Staphylococcus*, *Salmonella, E. coli,* and *Shigella.* Interestingly, some of these organisms are natural habitants in the intestines of humans and animals [5, 9]. The presence of bacteria in milk products constitute a public health hazard [9]. Contamination of milk occurs either during milking from the udder, milking personnel and milking equipment, or from transportation and storage of the milk post-milking. Under any of these conditions, microorganisms get into the milk and multiply. The distance between the farm, the processing plant and the consumers, the time lapsed during transportation, and the temperature of milk during storage gives bacteria the chance to adapt and grow in this nutritious liquid, thereby increasing the microbial load [10]. Contaminated raw milk affects the whole dairy industry, ultimately resulting in milk with either poor manufacturing properties or dairy products with reduced flavor quality, as well as reduced shelf life [11]. The importance of milk quality cannot be overemphasized; hence, it is germane to ensure that high-quality raw milk is produced from healthy animals under hygienic conditions and that control measures are applied to protect human health. The current study seeks to evaluate microbial milk quality along the value chain at AU Farm.

## 2. Materials and Methods

### 2.1. Study Site

The experiment was carried out at Africa University Farm located in old Mutare, Zimbabwe. It has an annual average summer temperature of 19 °C ranging from 16°C to 39°C and winter of 13°C ranging from 6°C to 20°C. The area lies at an latitude of S180 53.786 and a longitude of *E* 0320 36.036 and at an altitude of 1104 meters above the sea level (masl). It receives rainfall above 800 mm/annum and consists of three soil types; sand clay, loamy, sandy clay, and sand soil.

### 2.2. Experimental Design

A cross-sectional study design was formulated to determine the source of bacterial spoilage of milk, with the collection stage (during milking, bulk tank storage, and DH) and weeks of collection as treatments in a completely randomized design (CRD). Raw samples were collected once per week for three consecutive weeks.

### 2.3. Animal Management

A total of eighteen lactating Holstein-Friesian cows, 450 ± 30 kg live weight between 60–100 days of lactation, in their second parity, were used for the experiment. The selected cows had an average body condition score of three and in good health. Machine milking was done twice per day in the morning (0600–0800) and in the afternoon (1400–1600 hrs). The cows were managed under a semi-intensive production system whereby they were grazed in confined paddocks dominated by *Eragrostis, Sporobolus*, and *Panicum* species, then received a dairy concentrate supplement (Midlak 18% Dairy Meal) at a rate of 1.5 to 2% of their live weight. Ten days before the experiment, the cows were dewormed against internal parasites using Valbazen (Zoetis, SA) and sprayed against ticks using Triatix dip 12.5% (Coopers) once every week.

### 2.4. Sample Collection

Prior to milking, samples were collected after the udder was cleaned and ready for milking. The cleaning process included, among other things, a gentle wash under running water, teat dipping (Dairi Teat Dip, Iodine 1.5% Chemplex), and mastitis check and drying using a disposable paper. Bulk containers and milking containers at the farm and at the dining hall (DH) were rinsed with 100 ml sterile water prior to random sample collection into sterile sampling bottles. Raw milk samples were taken directly from either the bulk tank or DH containers with the aid of a ladle, which was previously disinfected with approximately 70% alcohol according to Marcondes et al. [12]. A cold chain (4°C) was maintained in all storage facilities and sampling was done ±3 hours post milking. A total of two hundred and seventy milk samples, approximately 5 ml, were aseptically collected at each stage; 162 for milking, 54 from the bulk tank, and 54 from DH and put into sterile milk vials over a period of 21 days. The collected samples were stored at 4°C for at most twenty-four hours before being subjected to microbial analysis which was done in the Food Science Laboratory, Department of Agriculture at AU.

### 2.5. Bacteriological Analysis

The bacteriological tests of TBC, *Salmonella enterica,* and *E. coli* were determined to indicate the bacterial load in raw milk samples at different stages along the milk chain.

### 2.6. Serial Dilution, Culturing, and Enumeration of Microorganisms

Approximately, 5 ml of milk was transferred to 4.5 ml sterile peptone water solution and mixed thoroughly to make 10^−1^ dilution. After which, serial dilutions of 0.5 ml were then transferred to another 4.5 ml sterile test tube to make 10^−2^. This procedure was repeated to make four dilutions (10^−1^ to10^−4^). Using a sterile tip, 1 ml from each of the serial dilutions was aseptically transferred into sterile Petri plates; this was followed by the addition of 10–15 ml of the desired medium (molten nutrient agar for TBC and lauryl sulphate agar for *E*. *coli*) and replicated three times. Molten nutrient agar, which had been prepared and maintained in a water bath at 56°C, was then added, mixed well, and allowed to solidify. The Petri dishes with molten nutrient agar were incubated at 32°C for 48 hours while the dishes with lauryl sulphate agar were incubated at 44°C for 48 hrs. The enumeration of total viable bacteria was done according to the methodology of Houghtby et al. [13]. Only Petri dishes with colonies ranging from 30–300 were selected for counting and a colony counter was used (Scan 300, Saint Nom, France**)**. The number of colonies in each dilution was multiplied by the reciprocal of the dilution and recorded as colony forming units (cfu/ml). All samples positive for *E. coli and S*. *enterica* contamination were confirmed using Gram's staining, cultural biochemical examinations, and sugar fermentation tests (Table 1). The biochemical tests performed included catalase, citrate, indole, methyl red, nitrate reduction, urease production, and motility tests.

### 2.7. Statistical Analysis

ANOVA GLM of SPSS version 16 software was used for data analysis. The following model was used:

(1) $$\begin{array}{c} \text{Y ijk }=\;µ+\text{ Si }+\text{Tj }+\text{ ejk}, \end{array}$$

where *y* = response variable (TBC, *E coli*, *S. enterica*) and *µ* = mean common to all variables. Si = treatment effect (i; post milking storage, DH).. Tj = effect of the week of collection (*j* = 1, 2, 3).. Ej = error term.

Means were separated using the Tukey HSD test.

## 3. Results

The biochemical test results for *E. coli* and *S*. *enterica* are shown in Table 1. Both species were positive for catalase, nitrate, and methyl red while able to ferment glucose and mannitol. *Escherichia coli* was also able to ferment lactose, salicin, and sucrose while *S. enterica* could not.

The mean values for microbiological quality of raw milk samples collected from different sites along the value chain are summarized in Table 2.

**Figure 1:**
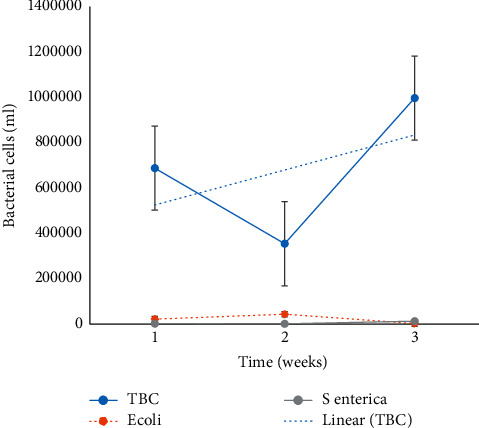
TBC, *E coli*, and S *enterica* cells from raw milk.

TBC and *E*. *coli* did not show a consistent pattern among collection sites and weeks, while *S. enterica* increased significantly (*P* < 0.05) from one site to the next. TBC increased (*P* < 0.05) from one site to the next along the value chain, yet it undulates when measured over time. *Escherichia*. *Coli* increased (*P* < 0.05) along the chain and the last site of collection and the greatest population recorded in week 2. The TBC, *E*. *coli*, and *S. enterica* populations increased (*P* < 0.05) from milking to storage at the DH. Measured over time, TBC increased (*P* < 0.05) from week 1 to week 3, while *E*. *coli* significantly (*P* < 0.05) increased in the second week, recording the highest bacterial population (Figure 1) and *S. enterica* did not show any pattern.

## 4. Discussion

Milk quality is perceived differently by different groups of people, the focus should be aimed at prevention of defects, rather than their detection [14]. *Escherichia*. *coli* and *S. enterica* tested positive for catalase, nitrate reduction, and methyl red. A positive result for catalase suggests that the bacterium possesses hemolytic enzymes, which when provided by a red blood cell enriched culture medium would destroy the cells and or digest the hemoglobin [15]. The ability of bacteria to convert nitrate to nitrite in mammals has been coined to be important in humans with dental caries [16]. The lower the bacteria load, the better the quality of the milk [17]. The overall mean TBC in the current study was lower than the recommended standard of 2.0 × 10^6^ cfu/ml [18, 19] and that reported by Mhone et al. [20] for Zimbabwe (6.4 log10 cfu/mL); van Schaik et al. [21] for the United States (4.06 log10 cfu/mL); Ruusunen et al. [22] for Finland (4.11log10 cfu/mL); or Tamirat [23] for Ethiopia (6.15log10 cfu/ml). This indicates that milk produced at AU farm is of higher microbial quality. The TBC is an appropriate estimate of good quality raw milk [24] and has become one of the accepted criteria for grading milk. In agreement, Oliver et al. [25] reported that high-quality raw milk has a low TBC. The Pasteurized Milk Ordinance of the United States instigates that grade A raw milk should have a TBC of <1 × 10^5^ cfu/mL [26], similar levels are also authorized in Europe [27]; however, in China, the national standard accepts a higher TBC of <2 × 10^6^ cfu/mL [28]. It is generally accepted that the frequency of milking significantly affects TBC counts, such that herds milked 3 times/d show a lower TBC level than herds milked 2 times/d. Results from the current study are contrary to this phenomenon since the animals were milked 2 times/d but still showed lower TBC counts. There were significant differences in TBC, *E*. *coli,* and *S. enterica* at different sites and times of collection. The increase in TBC count from milking to final storage at the dining hall was not expected. The main reason for this disparity emanated from utensils that are used during milk transportation and failure to maintain a cold chain in transit [29]. This supports earlier reports by Wallace (2008) who highlighted that milk holding temperature and the length of storage prior to testing and processing allow bacterial milk contamination. In support, Jay et al. [30] reported that *Salmonella species* can survive long periods of storage temperatures < −20 C. As evidenced by a sharp increase in both *E*. *coli* and *S. enterica,* the utensils were dirty and this increased the TBC [24]. Mohamed et al. [31] also reported that *Salmonella* spp., which can grow in improperly stored raw milk and milk products, presents a public health risk. All this only serves to confirm the five critical control points identified by Ndungu et al. [32]. These include milking at the farm level, bulking milk at collection points, transportation, and at the cooling tank.

The consumption of pathogen-containing milk has been reported to cause illnesses ranging from stomach upset to more serious symptoms, for example; diarrhea, fever, and vomiting [33]. According to Al-Khatib and Al-Mitwalli [34], gastroenteritis, diarrhea, typhoid, or bovine *tuberculosis* are the main life-threatening disease conditions associated with milk.

Other milk bacteria, although not determined in the current study, may potentially affect the products' nutritional and sensory quality properties; this in turn results in significant economic losses. Furthermore, the presence of *E. coli* and *S. enterica* has been reported to cause severe illnesses in humans and other food-borne illnesses [35, 36]. For example, *S. enterica* (formerly *Salmonella typhimurium)* was proposed to cause over 99% of severe human food-borne illnesses [39]. Interestingly, *Salmonella* enterotoxin is closely related to the cholera toxin functionally, immunologically, and genetically as well as the heat-labile toxin of pathogenic *E. coli* [30]. However, the two species cause illness in a different way; diarrhea associated with heat labile *Salmonella* enterotoxin is released during species growth, which results in the loss of intestinal fluids (damage of the intestinal mucosal). While heat-labile *E. coli* interferes with water and electrolyte movement across the intestinal epithelia [40], by so doing the volume of accumulated fluid exceeds the normal absorptive capacity of the large intestine leading to watery diarrhea.

The current study also indicates hygiene of the dairy parlor with very low bacterial counts during bulk tank storage. The relationship between TBC, *E. coli*, and *S. enterica* is nonlinear with respect to time; it actually appears negative in the second week of collection, and Lan et al. [24] reported a similar outcome. The reason for the undulations could be attributed to the use of different dairy men on a weekly basis. The human factor and unhygienic conditions [39] are the greatest causes for milk and milk product contamination. Furthermore, *S. aureus* and *E. coli* have been reported to significantly increase TBC counts [24].

The overall mean for *E. coli* of 6.0 × 10^5^ cfus/ml recorded at DH was higher than the maximum recommended level of 5.0 x 10^4^ cfu/ml (EAS 67 : 2006). Again, this was not expected since *E. coli* is an indicator of mainly fecal bacterial contamination [39, 40]. Increases in *E. coli* could theoretically be attributed to coliform mastitis, as mastitic cows have been shown to shed it levels as high as 10^8^ cfu/ml (16) (Hayes et al. 2001).

*E*. *coli* causes deadly diarrhea in humans who more often than not obtain it from the consumption of contaminated raw milk [41]. Generally, the findings observed in the current study showed that the animal house is too close to the dairy parlor and this could be a ready source of contaminating bacteria. The major problem facing the dairy industry in Zimbabwe is to ensure the production of the quality of raw milk which can easily be processed into milk products. The level of *Salmonella* in our study were lower comparable with that of Malaysia and United States [42], but higher than that from New Zealand [43]. Evidence from the current study shows that contamination of milk is generally after milking, this was also reported by Mohamed et al. [31] in Djibouti. The emergence of antibiotic resistant strains is the recent worlds' recent concern to this end *Salmonella* spp., particularly *S. typhimurium* definitive phage type 104 (DT104) which is a significant human and animal pathogen is on the lookout. The species is known for its high morbidity and which has been reported in cattle and poultry products [36].

## 5. Conclusion

In conclusion, we found that raw milk samples met the TBC, *E*. *coli,* and *S*. *enterica* standards of Zimbabwe during milking and in the bulk tank; thus, the animals were not the source of microbial contamination. Bacterial load increased during transit and storage and at the DH, suggestive of the fact that the DH personnel/equipment could be the source of contamination. Therefore, there is a need to improve the degree of cleanliness and hygiene post milking.

## Figures and Tables

**Table 1:** Biochemical reactions for *E*. *coli and S*. *enterica*.

Biochemical tests	*E. coli*	*S. enterica*
Catalase	+ve	+ve
Citrate	-ve	+ve
Indole production	+ve	-ve
Nitrate reduction	+ve	+ve
Methyl red	+ve	+ve
Urease	-ve	-ve
Motility	+ve	+ve

*Acid sugar/Fermentation of*		
Glucose	+ve	+ve
Mannitol	+ve	+ve
Lactose	+ve	-ve
Salicin	+ve	-ve
Sucrose	+ve	-ve

**Table 2:** LS means of TBC (x1000 cfu/mL) *E. coli* (x1000 cfu/mL) and *S. enterica* (x1000 cfu/mL) of raw milk produced at AU Farm.

	*Collection stage (S)*				*Time (T) (weeks)*			SE	*P values*		
	DM	BT	DH	SE	1	2	3		S	T	ST
TBC	913.4^c^	919.3^b^	1160.5^a^	106	689.9^b^	356.5^c^	2568.8^a^	106	0.001	0.001	0.002
*E. coli*	4.4^b^	2.3^b^	60.4^a^	18.1	21.4^b^	43.5^a^	21.0^b^	18.1	0.003	0.022	0.022
*S. enterica*	19.66^c^	55.00^b^	80.33^a^	2.33	16.33^a^	8.33^c^	12.67^b^	2.33	0.001	0.003	0.09

^abc^ means with different superscripts are significant at (*P* < 0.05), SE = standard error DM = milking, BT = bulk storage, DH = dining hall.

## Data Availability

The data used to support the findings of the study will be made available on request through the corresponding author.
